# Body mass index ≥23 is a risk factor for insulin resistance and diabetes in Japanese people: A brief report

**DOI:** 10.1371/journal.pone.0201052

**Published:** 2018-07-20

**Authors:** Tsuyoshi Okura, Risa Nakamura, Yohei Fujioka, Sonoko Kawamoto-Kitao, Yuichi Ito, Kazuhisa Matsumoto, Kyoko Shoji, Keisuke Sumi, Kazuhiko Matsuzawa, Shoichiro Izawa, Etsuko Ueta, Masahiko Kato, Takeshi Imamura, Shin-ichi Taniguchi, Kazuhiro Yamamoto

**Affiliations:** 1 Division of Cardiovascular Medicine, Endocrinology and Metabolism, Tottori University Faculty of Medicine, Yonago, Tottori, Japan; 2 School of Health Science, Tottori University Faculty of Medicine, Yonago, Tottori, Japan; 3 Division of Molecular Pharmacology, Tottori University Faculty of Medicine, Yonago, Tottori, Japan; 4 Department of Regional Medicine, Tottori University Faculty of Medicine, Yonago, Tottori, Japan; University of Colorado Denver School of Medicine, UNITED STATES

## Abstract

**Background:**

Screening for undiagnosed type 2 diabetes mellitus is recommended for Asian Americans with a body mass index ≥23. However, the optimal body mass index cut-off score for predicting the risk of diabetes mellitus in Japanese people is not well known. The aim of this study was to determine the best body mass index cut-off score for predicting insulin resistance and diabetes mellitus in the Japanese population.

**Methods:**

This study had two parts, a clinical investigation and a retrospective observational investigation. In the clinical part of the study, 58 participants (26 with type 2 diabetes mellitus and 32 non-diabetics) underwent a hyperinsulinemic-euglycemic clamp from which their glucose disposal rate was measured. For the retrospective part of the study, medical check-up data from 88,305 people in the Tottori Prefecture were analyzed for clinical evidence of diabetes mellitus. The optimal BMI cut-off scores for prediction of insulin resistance and diabetes mellitus were determined.

**Results:**

In the clamp study, the optimal body mass index cut-off score to predict insulin resistance in non-diabetic patients was 22.7. All participants with type 2 diabetes mellitus were insulin resistant, and the optimal body mass index cut-off score for prediction of severe insulin resistance was 26.2. When the data from the type 2 diabetic and the non-diabetic participants were combined, the optimal body mass index cut-off score for prediction of insulin resistance was 23.5. Analysis of 88,305 medical check-up records yielded an optimal body mass index cut-off score for prediction of diabetes mellitus of 23.6.

**Conclusions:**

These results suggest that having a body mass index ≥23 is a risk factor for insulin resistance and diabetes mellitus in the Japanese population.

## Introduction

The pathophysiology of type 2 diabetes mellitus (T2DM) involves insulin resistance and impaired insulin secretion [1]. Obesity is a condition strongly associated with the development of insulin resistance and T2DM. The World Health Organization (WHO) defines obesity as a body mass index (BMI) >30 and overweight as a BMI of 25–30 [2]. The Japan Society for the Study of Obesity defines obesity as a BMI ≥25 [3]. Therefore, the risk of insulin resistance is an important consideration for those with a BMI ≥25. However, a WHO panel reported that Asian populations have a lower BMI cut-off point for T2DM and cardiovascular disease risk than the WHO criteria of BMI 25 [4]. The American Diabetes Association recently recommended that testing for diabetes should be considered for all Asian American adults who present with a BMI of ≥23 [5]. However, the BMI range for overweight has not been defined for Japanese people, and there are no guidelines that define optimal BMI cut-offs for predicting diabetes in the Japanese population.

The most precise method for assessing insulin resistance is the hyperinsulinemic-euglycemic clamp test; however, this test is very complicated [6]. Instead, the homeostasis model assessment for insulin resistance (HOMA-IR) is widely used in clinical practice and in clinical studies [7]. However, the reliability of HOMA-IR is limited in the patients with low BMI values, decreased beta-cell function, and/or high fasting glucose levels [8]. Since Asian and Japanese patients often have decreased beta-cell function [9], a clamp study is required for accurate evaluation of insulin resistance in these populations.

In this study, we aimed to determine the optimal BMI cut-off score for prediction of insulin resistance and diabetes mellitus (DM) in Japanese people using a glucose clamp test in patients with T2DM and in non-diabetic volunteers. We also analyzed the prevalence of diabetes in approximately 90,000 participants in Tottori Prefecture using data from medical check-ups.

## Research design and methods

### Subjects

This study consisted of a clinical investigation and a retrospective analysis of medical check-up records. A total of 58 volunteers participated in the clinical investigation. Twenty-six of these were patients treated at the Tottori University Hospital between 2014 and 2017 who were diagnosed with T2DM using the WHO criteria [10]. Prospective participants with pancreatic disease, liver disease, or renal failure, and those taking diabetogenic medications such as corticosteroids, were excluded from this study. All participants in the T2DM group were on diet therapy alone and were not taking any medications aimed at treating their T2DM. Thirty-two non-diabetic volunteers (Non-DM group) also participated in this study. The Non-DM participants were residents of Tottori Prefecture who received a routine medical check-up at our institute; they were recruited to participate in the glucose clamp study by written request after their check-up showed that they did not have, and were not taking medication for, T2DM, hypertension, or dyslipidemia. This study was conducted according to the principles expressed in the Declaration of Helsinki, and approved by the Ethics Committee of the Faculty of Medicine at Tottori University (approval number G161). Written informed consent was obtained from all of the participants prior to their participation in this study.

### Hyperinsulinemic-euglycemic clamp

We performed glucose clamp tests as previously reported [11]. Briefly, the hyperinsulinemic-euglycemic clamp was performed using an artificial endocrine pancreas (STG 55; Nikkiso, Shizuoka, Japan) to evaluate insulin sensitivity [1]. A primed constant infusion of insulin (100 mU/m^2^/min) was administered to each participant and plasma glucose levels were maintained at 5.2 mmol/L (95 mg/dL). According to previous studies, this method achieves a steady-state plasma insulin level of 1200 pmol/L in patients with T2DM [12, 13]. The steady-state glucose infusion rate (GIR) from 90–120 min after the start of the infusion. The mean GIR during this time was defined as the glucose disposal rate (GDR), which was used as a marker of peripheral insulin sensitivity. The glucose clamp method is a well-established procedure at our hospital [11, 14].

At the insulin infusion rate used in this protocol, we defined GDR >10 mg/kg/min as normal, GDR <10 mg/kg/min as insulin resistance [15, 16, 17], and GDR <5 mg/kg/min as severe insulin resistance [18].

### Calculation of insulin resistance indices

Insulin resistance was calculated as follows: HOMA-IR [8] = [fasting plasma glucose (mmol/L)] × [fasting plasma insulin (pmol/L)]/135.

### DM prevalence in patients undergoing medical check-ups in Tottori Prefecture

We detected the prevalence of DM in Tottori Prefecture using medical check-up data from patients in this region in 2013. In diabetic patients, the characteristics of the diabetes were not always indicated in the portion of the record that we examined; therefore, patients were classified as having DM or not but were not specifically classified as having T2DM. This retrospective, observational, epidemiological study was approved by the Insurer Council of Tottori Prefecture, who gave their written informed consent allowing use of their anonymized data. No personal identifying information was present in any of the medical check-up records used. According to the Japanese Ethical Guidelines for Epidemiological Research, informed consent does not necessarily need to be obtained from research subjects in observational studies using only existing materials, particularly when subjects are not identifiable in the records used [19]. DM was defined as follows: fasting plasma glucose (FPG) ≥126 mg/dl or glycated hemoglobin (HbA1c) ≥6.5%, or (because not all participants were assessed for FPG and HbA1c) a history of treatment for DM. The numbers of patients assessed for each measure were: FPG, not HbA1c, 45,411; HbA1c, not FPG, 18,314; both FPG and HbA1c, 22,851.

### Statistical analysis

Data are expressed as means ± standard deviation of the mean. Differences in mean values of characteristics between T2DM and Non-DM participants were assessed using unpaired t-tests. We determined BMI cut-off values using receiver operating characteristic (ROC) analysis. To evaluate the ability of BMI to predict insulin resistance in the clamp study, and DM in the medical check-up study, we plotted ROC curves. Diagnostic properties of BMI cut-off scores were defined by maximizing the sensitivity and specificity to identify GDR <10 and the presence of DM. The optimal cut-off points were obtained using ROC curves and the Youden Index (maximum [sensitivity + specificity − 1]) [20]. The cut-off point on the ROC curve was closest to (0,1), and was calculated as the minimum value of the square root of ([1 − sensitivity]^2^ + [1 − specificity]^2^) [21]. Values were considered statistically significant if *P* <0.05. SPSS software version 26.0 (IBM Corp., Armonk, NY, USA) was used for all analyses.

## Results

### Cut-off BMI scores for detection of insulin resistance determined by hyperinsulinemic-euglycemic clamp

The characteristics of the participants in the T2DM and Non-DM groups are shown in Table 1. Mean age, BMI, waist circumstance, FPG, HbA1c, and HOMA-IR were all significantly higher in the T2DM group than in the Non-DM group, and the mean GDR was significantly lower in the DM group than the non-DM group.

**Table 1:** Participant characteristics in the glucose clamp study.

	Total	T2DM	Non-DM	P value
*n*	58	26	32	
Sex (male/female)	25/23	16/10	19/13	
Age (years)	41.9 ± 14.9	54.0 ± 12.0	32.1 ± 8.3	<0.001
BMI (kg/m^2^)	24.0 ± 4.4	26.9 ± 4.1	21.8 ± 2.9	<0.001
Waist circumference (cm)	85.8 ± 13.7	94.3 ± 11.0	76.2 ± 9.8	<0.001
Fasting plasma glucose (mmol/L)	5.6 ± 1.6	6.9 ± 1.0	4.5 ± 1.2	<0.001
HbA1c (%)	6.3 ± 1.2	7.4 ± 0.9	5.3 ± 0.3	<0.001
HbA1c (mmol/mol)	45 ± 58.5	57 ± 10	34 ± 4	<0.001
HOMA-IR	2.4 ± 2.0	3.5 ± 2.7	1.5 ± 1.0	<0.001
GDR (mg/kg/min)	7.9 ± 3.0	5.7 ± 2.1	9.6 ± 2.5	<0.001

Data are means ± standard deviation. P-values were determined using unpaired t-tests, T2DM vs. non-DM. BMI, body mass index; GDR, glucose disposal rate; HbA1c, glycated hemoglobin; HOMA-IR, homeostasis model assessment for insulin resistance; Non-DM, non-diabetic study participants; T2DM, study participants with type 2 diabetes mellitus

**Fig 1:**
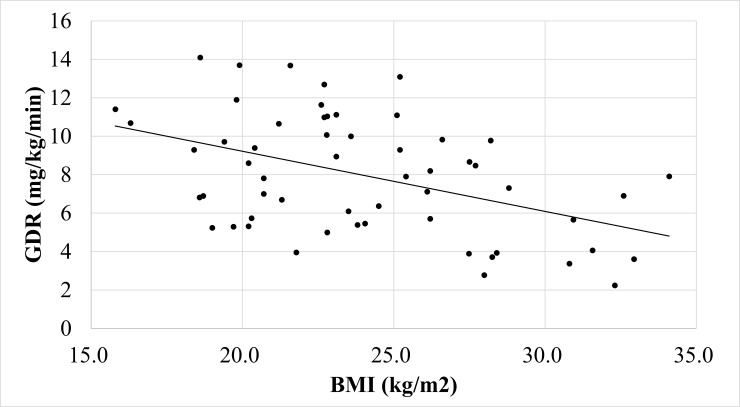
The correlation between BMI and GDR in the all participants in the clamp study.

**Fig 2:**
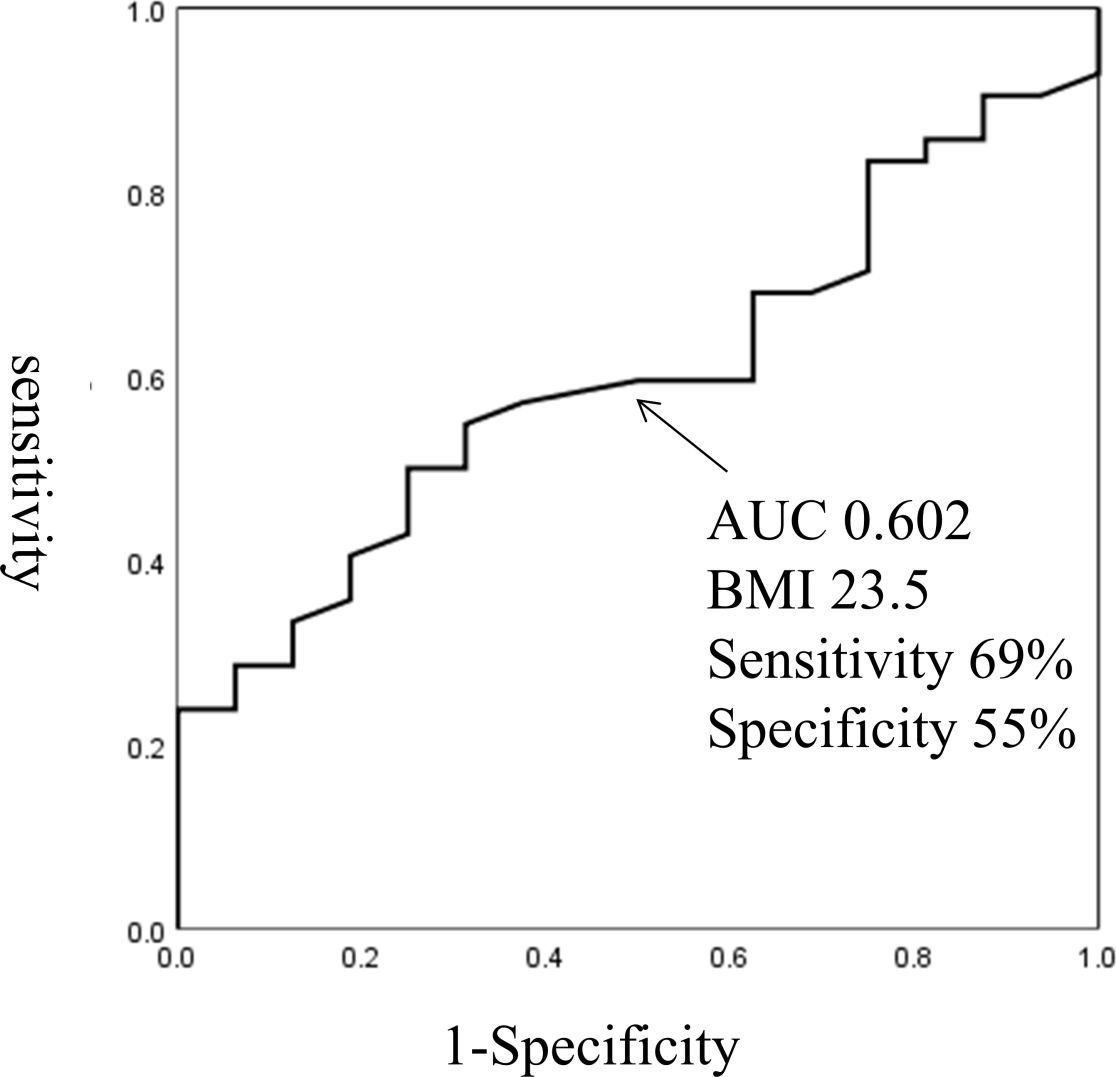
The optimal BMI cut-off score for prediction of insulin resistance in all participants in the T2DM and Non-DM groups combined.

**Fig 3:**
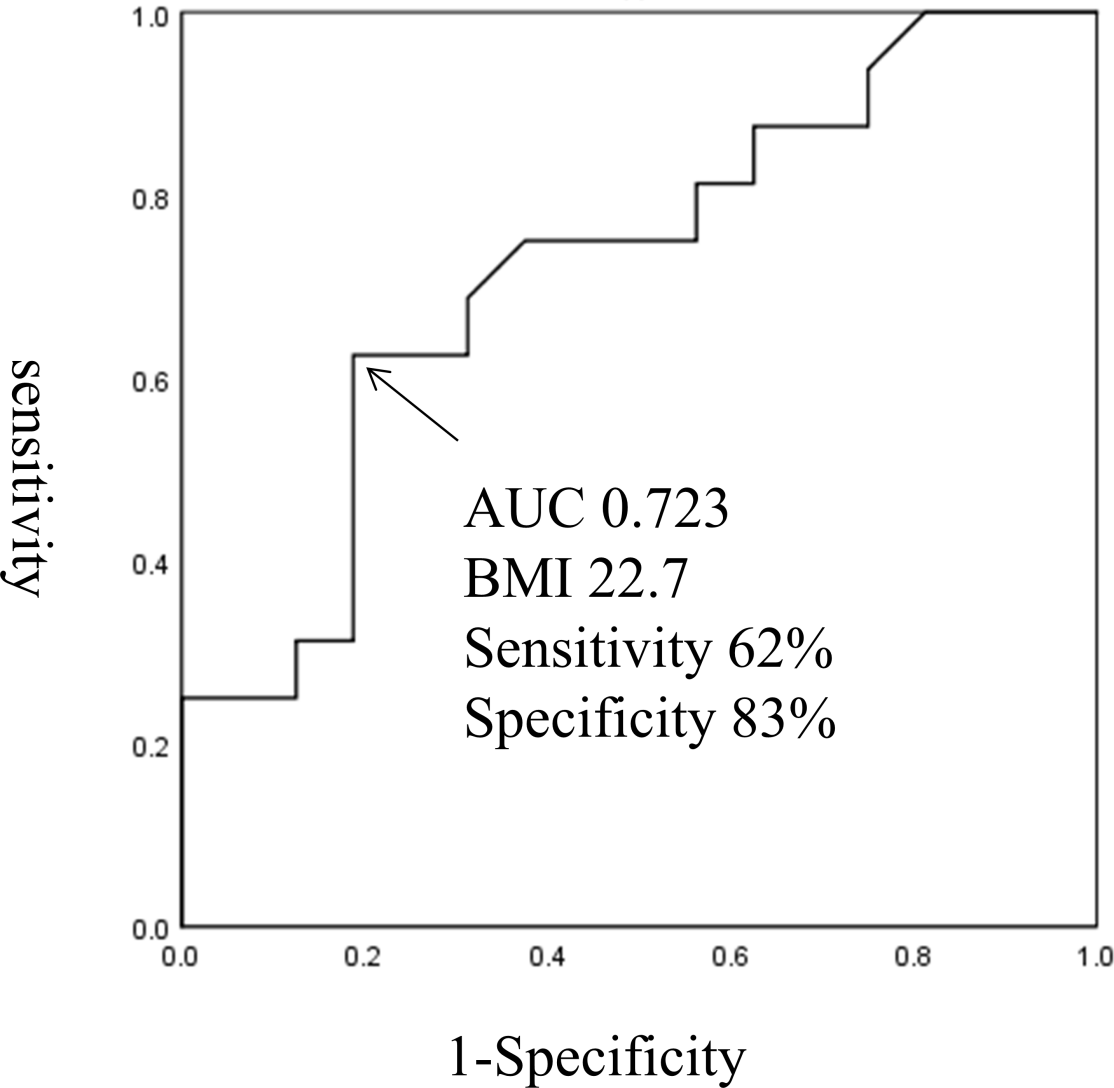
The optimal BMI cut-off score for prediction of insulin resistance in the Non-DM participants.

**Fig 4:**
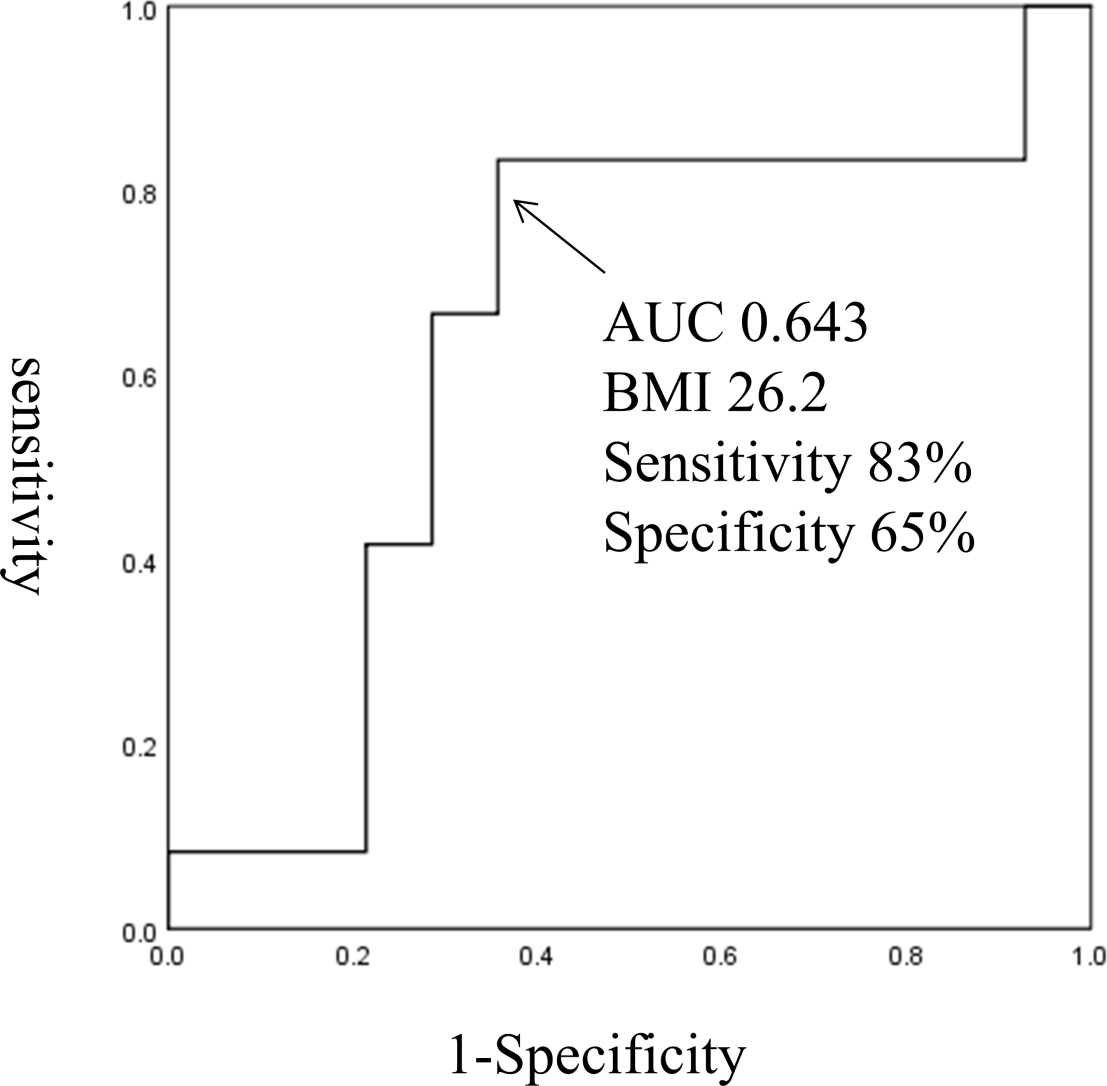
The optimal BMI cut-off score for prediction of severe insulin resistance in the T2DM group.

BMI was significantly correlated with GDR (R = 0.67, P<0.0001; Fig 1). Of the 58 participants in the clamp study, 42 were insulin resistant (defined as having a GDR <10). The optimal BMI cut-off score for prediction of insulin resistance was 23.5 (69% sensitivity, 55% specificity, AUC 0.602; Fig 2). Of the 32 Non-DM participants, 16 had insulin resistance, defined as GDR <10. The optimal BMI cut-off score to predict insulin resistance in the Non-DM participants was 22.7 (62% sensitivity, 83% specificity, AUC 0.723; Fig 3). Of the 26 T2DM participants, all had insulin resistance and 12 had severe insulin resistance defined as GDR <5.0. The optimal BMI cut-off score to predict severe insulin resistance in the T2DM group was 26.2 (83% sensitivity, 65% specificity, AUC 0.643; Fig 4).

### Cut-off BMI scores for detection of DM determined from analysis of medical check-up records

We analyzed the medical check-up records of 88,305 people in Tottori Prefecture and detected 8,311 people with T2DM (Table 2). The mean age of people with DM was 62.7 years, and mean BMI was 22.8 kg/m^2^, 5,229 had a history of DM treatment, 2,231 had FPG ≥126 mg/dl, 1,303 had HbA1c ≥6.5%, and 452 had FPG ≥126 mg/dl and HbA1c ≥6.5%.

**Table 2:** Participant characteristics in the medical check-up study.

	Total	DM	Non-DM	P value
*n*	88,305	8,316	79,989	
Sex (male/female)	41,518/46,787	5,116/3,200	36,402/43,587	
Age (years)	62.7 ± 13.3	67.0 ± 11.4	62.3 ± 13.4	<0.001
BMI (kg/m^2^)	22.8 ± 3.3	24.2 ± 3.8	22.6 ± 3.2	<0.001
Waist circumference (cm)	82.1 ± 9.5	87.3 ± 10.1	81.6 ± 9.3	<0.001
Fasting plasma glucose (mmol/L)	5.3 ± 1.4	7.7 ± 2.3	5.1 ± 1.0	<0.001
HbA1c (%)	5.5 ± 1.2	6.9 ± 2.0	5.4 ± 1.0	<0.001
HbA1c (mmol/mol)	37 ± 13	52 ± 22	36 ± 10	<0.001

Data are means ± standard deviation. BMI, body mass index; HbA1c, glycated hemoglobin; DM, study participants with diabetes mellitus; Non-DM, non-diabetic study participants. P-values were determined using unpaired t-tests, DM vs. non-DM.

**Fig 5:**
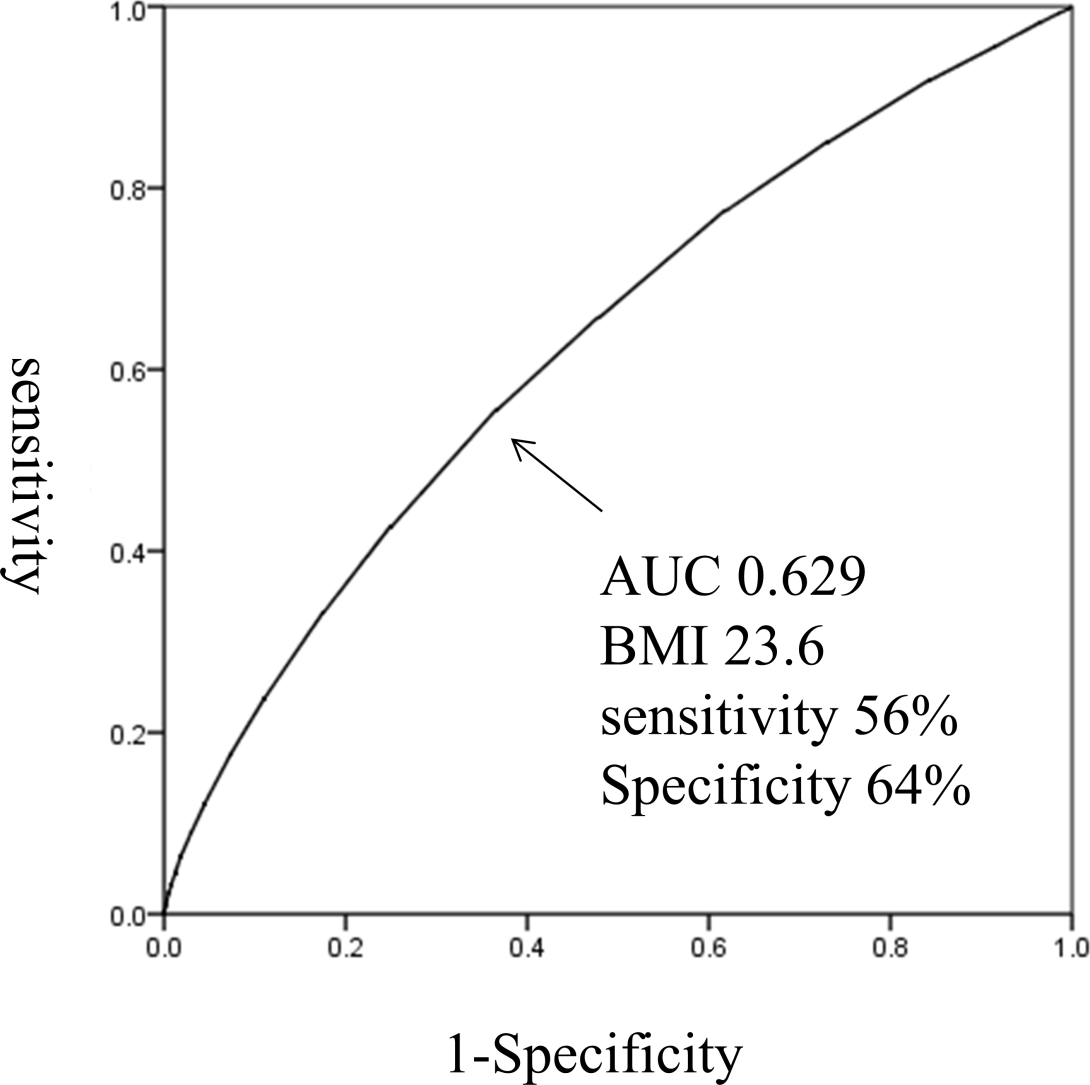
The optimal BMI cut-off for detection of DM in patient in Tottori Prefecture.

The optimal BMI cut-off score for detection of DM in this population was 23.6 (56% sensitivity, 64% specificity, AUC 0.629; Fig 5).

## Discussion

The results of this study indicate that the optimal BMI cut-off score for prediction of insulin resistance in all of the Non-DM and T2DM participants in the glucose clamp study was 23.5. The optimal BMI cut-off score for prediction of insulin resistance in Non-DM participants using the glucose clamp method was 22.7. In the analysis of medical check-up data, the optimal BMI cut-off score to predict DM was 23.6. These results suggest that having a BMI ≥23 is a risk factor for insulin resistance and DM in the Japanese population. This is consistent with the recommendation of the American Diabetes Association that testing for diabetes should be considered for all Asian American adults who present with a BMI of ≥23 [5]. A recent study also suggested that, among Japanese men with BMI ranging from 23–25, muscle insulin resistance was present in those with even one cardiometabolic risk factor (hypertension, hyperglycemia, or dyslipidemia) [22]. Because the Non-DM participants in our study did not have hypertension, hyperglycemia, or dyslipidemia, our results show that there is a risk of insulin resistance in Japanese people who have a BMI ≥23 even without the presence of cardiometabolic risk factors. In addition, all T2DM participants in the clamp study were insulin resistant, and the BMI cut-off score for detecting severe insulin resistance was 26.2. These results suggest that most Japanese people with T2DM are insulin resistant and that having a BMI >25 is a risk factor for severe insulin resistance. These results have important implications for clinical practice in Japan.

There are few Japanese population-based reports of BMI cut-off scores for predicting diabetes diagnosed by the oral glucose tolerance test (OGTT). The Japan Diabetes Complication Study showed that the mean BMI of 2,205 patients with T2DM was 23.1; however, this study did not perform OGTTs [23]. A pilot study reported that the optimal BMI cut-off value for prediction of worsening glucose metabolism diagnosed by OGTT was 23.1 kg/m^2^ in 604 Japanese participants [24]. These results and our results indicate that BMI >23 is a risk factor for diabetes in Japanese people. However, further studies using OGTTs and larger numbers of subjects are needed.

Our study had several limitations. The relatively small number of participants who underwent the glucose clamp and the differences in age and BMI between the T2DM and Non-DM groups indicate that our results require confirmation in a larger study. There are several important risk factors for insulin resistance that we were unable to control for in our study. These include age, body composition, and gender, all of which could have affected our BMI cut-off analyses. In our clamp study there was no significant difference in the sex ratio between the T2DM and Non-DM group; however, participants in the T2DM group were significantly older than those in the Non-DM group. It is possible that this could have influenced our results. However, it has been previously shown that, when age is taken into account, BMI is still a significant risk factor for T2DM [25]. Thus, we feel that our BMI cut-off values are still relevant even with our relatively small sample size. We are currently working on a larger study, the results of which we plan to publish in the future.

In the analysis of medical check-up records, it was not always possible to determine the type of DM (type 1, type 2, or other) from the portion of the record that we had access to. However, type 1 DM is rare in the Japanese population; the prevalence rate in Japan has been reported to be 10–15 per 100,000 [26]. Therefore, most participants with diabetes can be assumed to have T2DM. Moreover, a subset of participants with diabetes was diagnosed using FPG or HbA1c, and the AUC value of ROC analysis was low. Because there was no information on medication use in the records that we analyzed, it is possible that the use of medications such as insulin or insulin-sensitizers may have affected our study outcome. It is also important to consider other confounding factors such as age, body composition, and gender differences. However, despite these limitations we consider the results generated from this population-based analysis with a large number of participants to be meaningful. In the future, we plan to build on these results by conducting a population-based OGTT study. Despite its limitations, we believe that our study contributes information that may be important to screening strategies for insulin resistance and diabetes in the Japanese population.

In summary, this study showed that the optimal BMI cut-off score for prediction of insulin resistance in Non-DM participants diagnosed using a glucose clamp was 22.7. For the Non-DM and T2DM participants combined, the optimal BMI cut-off score for prediction of insulin resistance was 23.5. In the population-based medical record analysis, the optimal BMI cut-off score for prediction of DM was 23.6. These results suggest that having a BMI ≥23 is a risk factor for insulin resistance and DM in the Japanese population.
